# Human Papillomavirus Infection Is Associated with Decreased Risk of Hepatocellular Carcinoma in Chronic Hepatitis C Patients: Taiwan Nationwide Matched Cohort Study

**DOI:** 10.3390/cancers14051289

**Published:** 2022-03-02

**Authors:** Sung-Shuo Kao, Chia-Jung Li, James Cheng-Chung Wei, Cheng-Li Lin, Renin Chang, Yao-Min Hung

**Affiliations:** 1 Division of Gastroenterology and Hepatology, Department of Internal Medicine, Kaohsiung Veterans General Hospital, Kaohsiung 81362, Taiwan; kosuseki@vghks.gov.tw; 2 Kaohsiung Veterans General Hospital Pinging Branch, Pingtung 91245, Taiwan; 3 School of Medicine, College of Medicine, National Yang Ming Chiao Tung University, Taipei 11221, Taiwan; 4 Department of Obstetrics and Gynecology, Kaohsiung Veterans General Hospital, Kaohsiung 81362, Taiwan; nigel6761@gmail.com; 5 Institute of BioPharmaceutical Sciences, National Sun Yat-sen University, Kaohsiung 80424, Taiwan; 6 Institute of Medicine, Chung Shan Medical University, Taichung 40201, Taiwan; wei3228@gmail.com (J.C.-C.W.); rhapsody1881@gmail.com (R.C.); 7 Division of Allergy, Immunology and Rheumatology, Chung Shan Medical University Hospital, Taichung 40201, Taiwan; 8 Graduate Institute of Integrated Medicine, China Medical University, Taichung 40402, Taiwan; 9 Management Office for Health Data, China Medical University Hospital, Taichung 40402, Taiwan; orangechengli@gmail.com; 10 Department of Emergency Medicine, Kaohsiung Veterans General Hospital, Kaohsiung 81362, Taiwan; 11 Department of Internal Medicine, Kaohsiung Municipal United Hospital, Kaohsiung 80457, Taiwan; 12 College of Health and Nursing, Meiho University, Pingtung 91202, Taiwan

**Keywords:** human papillomavirus, HPV, hepatitis C virus, HCV, hepatocellular carcinoma, HCC, cohort

## Abstract

**Simple Summary:**

Previous studies have provided evidence suggesting a link between HCV and HPV-associated head and neck cancers. The epidemiological evidence of the relocated association between HPV and HCV-associated HCC is scarce. In the current study, from a secondary claim-based dataset, HPV infection is not associated with an increased risk of HCC in the CHC population. On the contrary, HPV infection seems to be associated with a lower risk of HCC development among patients with HCV infection. These findings suggest that the mechanism of association between procuring an HPV infection and reducing risk of HCC in the CHC population needs to be studied in detail in the future, with the opportunity to generate an intervention target that could delay the development of HCC.

**Abstract:**

Background: Hepatitis C virus (HCV) has been shown to be associated with human papillomavirus (HPV)-positive head and neck cancers. However, studies regarding HPV infection and the risk of new-onset hepatocellular carcinoma (HCC) among chronic hepatitis C (CHC) patients are limited. We examined the risk of HCC in CHC patients with or without HPV infection. Methods: In total, 9905 CHC patients from 2000 to 2016 constituted the whole cohort. HPV was defined as being diagnosed after HCV. The CHC cohort with HPV (*N* = 1981) and age-, sex-, inception point-, comorbidity-, and medication-matched non-HPV (*N* = 7924) were followed up until HCC, death, or 2018. HCC patients were extracted from the Taiwan Registry for Catastrophic Illness Database. We adopted the propensity score match and inverse probability of treatment weighting (IPTW) to eliminate bias. Cox proportional hazard regression analyses were performed to calculate HCC risk. Results: After a full adjustment, HPV was not associated with HCC risk (aHR, 0.74; 95% CI, 0.58–0.96 in the main model, and aHR, 0.76; 95% CI, 0.66–0.87 in IPTW, respectively). Almost all subgroup analyses verified this finding (HRs < 1.0). Conclusions: Among CHC patients older than 18 years old, those with HPV infection were associated with a lower risk of subsequent HCC.

## 1. Introduction

Hepatocellular carcinoma (HCC) is one of the most common cancers of the digestive system, with approximately 500,000 new cases diagnosed worldwide each year [1], and it also accounts for the second highest number of cancer deaths worldwide [2]. Therefore, the discovery of effective methods to prevent HCC is a critical public health issue. Hepatitis C virus (HCV) is a major contributor to the rising incidence of HCC worldwide and accounts for the second leading cause of HCC-related deaths [3]. Although progress in antiviral therapy has led to the dawn of HCV eradication and a reduced risk of HCC, the risk has not been completely eliminated [4]. The cirrhotic population appears to have an increased risk for HCC even after HCV eradication [5].

In ecosystems, there are simultaneous and rich heterogeneous interactions between microorganisms. Epidemiological and experimental analyses have provided quantitative evidence for the existence of subtle mutually beneficial or competitive interactions between respiratory viruses, especially cold and influenza viruses [6]. There are some more examples, such as HPV, which seems to be facilitated by human immunodeficiency virus (HIV) [7], whereas *Sta. aureus* is negatively associated with *Str. Pneumoniae* [8]. Pathogen–pathogen co-occurrence can lead to complicated competitive or co-operative forms of interactions.

The association of carcinogenesis between HPV and HCV has been mentioned [9,10]. HCV causes not only HCC, but has also been proposed to be linked to HPV-associated oropharyngeal head and neck malignancies [11]. HCV nonstructural protein _5_B (NS_5_B) recruit HPV oncoproteins E6, leading to the proteasomal degradation of retinoblastoma tumor suppressor protein (Rb) [12,13]. Thinking in the other direction, is there a potential interaction of HPV in the development of HCV-associated HCC? Both basic and epidemiological studies on this topic are scarce. We believe that it would be fruitful to examine the virus–virus interplay on the hard outcome HCC. Hence, the main objective of this retrospective secondary cohort study is to observe whether there is a synergistic or inhibitory effect of HPV infection in hepatocarcinogenesis in the CHC population.

## 2. Materials and Methods

### 2.1. Data Sources

After obtaining approval of institutional review board (IRB) from Taiwan Ministry of Health and Welfare, we identified medical records of all patients aged 20 years and older with hepatitis C infection collected in National Health Insurance Research Database (NHIRD). Taiwan’s National Health Insurance (NHI) program administered by the Taiwan government is single-payer and mandatory, and includes >99% of Taiwan’s population [14]. NHIRD is available for research purposes with appropriate application [15]. The validity of the NHIRD for use in epidemiological research studies has been shown in previous publication [10,16,17,18]. This study was a retrospective evaluation of patient information with no more than minimal risk to the subjects. It is impossible to identify individual patients, so informed consent was not required for this study. The encrypting procedure was identical and the linkage of claims belonging to the exact patient was constant and feasible for continuous follow-up. The NHIRD records were comprehensive and ongoing registration and claims information, including participants’ characteristics, disease diagnoses, outpatient visits, emergency department utility, and inpatient information, diagnostic, treatment, operation codes, and prescribed medications, were available. All claims could be linked in chronological order to provide a temporal sequence of all health service utilizations. Prior to 2016, the diagnosis codes used in this study were based on the International Classification of Diseases, Ninth Revision, Clinical Modification (ICD-9-CM), and from 2016 onwards, the diagnosis codes were based on ICD-10-CM. This study was approved by China Medical University Hospital Research Ethics Committee with certificated number CMUH109-REC2-031.

### 2.2. Identification of Study Sample (CHC Population)

The study database contained two million people, which was sampled by Bureau of National Health Insurance (BNHI) from the original claim data of NHIRD. The Health and Welfare Data Science Center (HWDC) of the Ministry of Health and Welfare provided data on the medical records and causes of death of 2 million sampled people to use. The frequently used variables in the NHI data and the cause of death data were provided directly for each application. The distributions of sex, age, and health care cost between the 2 million files and the 23 million population was similar. The National Health Research Institutes (NHRI) assigned a random number for each person by using the Knuth1 and Park and Miller’s 2 method [19,20].

There was no significant difference in the sex (*p* = 0.613) [21] and age distribution between the subset data and the original NHIRD [21]. We identified adult patients (>20 years) who had been newly diagnosed and medically recorded with HCV infection, based on the ICD-9-CM codes 070.41, 070.44, 070.51, 070.54, 070.70, 070.71, or V02.62, from 2000 to 2016 [16]. The diagnosis was verified by medical records review examined by board-certified gastrointestinal specialist for at least 3 outpatient visits or 1 hospitalization. A total of 10,800 CHCs were eligible for analysis. By this HCV definition, although exact proportion of patients with positive HCV RNA was unknown, the positive and negative predictive value could reach as high as 87% and 99%, respectively, and this was verified by a previous study linking NHIRD to New Taipei City Health Screening Database [22].

### 2.3. Exposure to HPV

Patients infected with HPV were extracted from the database according to ICD-9-CM codes 079.4, 078.1, 078.10-078.12, 078.19, 795.05, 795.09, 795.15, 795.19, 796.75, and 796.79. To avoid selection bias, the inclusion criteria required at least three outpatient visits or one inpatient diagnosis in the same database. This definition was used in our previous epidemiological studies [10]. The onset of HPV infection was defined as a diagnosis of HPV in the years 2000–2016, but not in the years 1997–1999. The inception point was the date of the first diagnosis of HPV in the time frame for the study group, and the date was assigned to the matched subjects in the control group. The control group comprised of CHC patients without a medical record of HPV before the inception point. To minimize misclassification, we also examined the control group to confirm that no HPV infection was diagnosed throughout the study period.

Of these patients, the testing of HPV infection was conducted by using a clinical physician’s judgement, but not by screening. There was no exact false negative rate of HPV infection. However, it is believed that this happens rarely because HPV testing has a great relationship with NHI payment for subsequent treatment and follow-up.

To increase the homogeneity, we excluded patients who had medical record of HBV infection (ICD-9 codes 070.22, 070.23, 070.32, 070.33, and V02.61), and viral hepatitis of others (ICD-9 codes 573.1, 573.2, and B190). Patients with malignancies (ICD-9-CM codes 140-208) were excluded prior to the index date to avoid potential liver metastases in our enrolled participants. Those that had HCC within 6 months after the inception day were excluded to avoid cases with under-diagnosed HCC. We excluded patients with missing information regarding age or sex.

### 2.4. Potential Confounders

Patients’ age and sex were recorded at the inception point. We also collected information about individual monthly income (<15,000 TWD or 540 USD, 15,000–29,999 TWD or 540–1080 USD, ≥30,000 TWD or 1080 USD), and urbanization level of residence area (1 to 4, 1 as the most urbanized and 4 as the least urbanized), a proxy for healthcare availability in Taiwan [23]. Baseline comorbidity was extracted from at least three consistent records in ambulatory care prior to the inception point. We identified the presence of following comorbidities using ICD-9-CM codes: hypertension (ICD-9-CM codes 401-405), diabetes (ICD-9-CM code 250), hyperlipidemia (ICD9-CM code 272), chronic kidney diseases (ICD-9-CM code 585), peptic ulcer disease (ICD-9-CM code 531-533), Helicobacter pylori infection (ICD-9-CM code 041.86), chronic obstructive pulmonary disease (ICD-9-CM codes 490-492 and 493-496), cirrhosis (ICD-9-CM codes 571.5, 571.6), liver decompensation (ICD-9-CM codes 571.5, 571.6, 572.2, 572.4), alcohol-related illness (ICD-9-CM codes 291, 303, 305, 571.1, 790.3, A215, V11.3), and major autoimmune diseases (ICD-9-CM codes 710.0, 714.x, 710.2). Medical confounders were identified by Anatomical Therapeutic Chemical codes. Aspirin, metformin, and statin were considered baseline drug therapy based on a minimum of 90 days of dispensing prescriptions for each drug in the 180 days prior to the inception point. Interferon usage was considered based on a minimum of 16 weeks of prescriptions at baseline.

### 2.5. Ascertainment of HCC Diagnoses and Follow-Up

We set new-onset HCC as the primary outcome of the study (ICD-9-CM code 155.0; ICD-10-CM codes C22.0, C22.7, C22.8). Patients with HCC were verified by linking to the Registry for Catastrophic Illness Patient Database (RCIPD). The positive predictive rate was 93% [24]. The study group and a 1:4 ratio of age-, sex-, comorbidity-, medication-, and starting point-matched controls were followed until the occurrence of HCC, emigration, imprisonment, withdrawal from NHI, or the end of the study (31 December 2018), whichever came first.

### 2.6. Matching Method

Baseline characteristics of participants were shown as event number and percentages for categorical variables and as means and standard. We used the propensity score method for analysis by referring to the following article by using the SAS program. This reference may help readers to more clearly understand the detailed statistical analysis procedures than log files of the statistical analyses from SAS [25].

The propensity score was calculated using the probability of the disease status assignment by using a logistic regression model, including the following baseline variables: age, gender, urbanization, individual monthly income (TWD); comorbidities of hypertension, diabetes, hyperlipidemia, chronic kidney disease, peptic ulcer disease, helicobacter pylori infection, COPD, cirrhosis, liver decompensation, alcohol-related illness, and autoimmune disease; every single reported medications of aspirin, metformin, statin, NSAIDs, interferon; index year.

CHC patients with HPV were matched (1:4 ratio) with those who did not have HPV according to their propensity score through nearest neighbor matching, initially to the eighth digit and then as required to the first digit. Therefore, matches were first determined within a caliper width of 0.0000001, and then the caliper width was increased for unmatched cases to 0.1. We reconsidered the matching criteria and performed a rematch (greedy algorithm). For each CHC patient with HPV, the corresponding comparisons were selected based on the nearest propensity score.

### 2.7. Statistical Analyses

Baseline characteristics of participants were shown as event number and percentages for categorical variables and as means and standard deviations for continuous variables. Chi-square test was adopted to examine the categorical variables, and Student’s *t* tests for continuous variables. All of the tests of significance were 2-tailed. *p*-value < 0.05 was considered statistically significant. The incidence of HCC during follow-up was calculated by dividing the number of events by the respective person years at risk and presented as the number of events per 1000 person years. Incidence rate ratio was assessed by Poisson regression. We plotted Kaplan–Meier curve to describe the cumulative incidence of HCC in the study and control group, and tested the difference between the groups by the log-rank test. We used multivariable Cox proportional hazards analysis to examine the HCC risk associated with HPV after adjustment for age, sex, hypertension, diabetes, hyperlipidemia, chronic kidney disease, peptic ulcer disease, Helicobacter pylori infection [26], COPD, cirrhosis, liver decompensation status, alcohol-related illness, CHC treatment, and medications. To validate the robustness of study findings (main model), four sensitivity analyses (model 2 to 5) were conducted.

### 2.8. Sensitivity Analyses

To address over-fitting issue, we conducted sensitivity analysis (Model 2) by matching for age, sex, index date, low individual monthly income, living area, comorbidity, and medications. Given the insidious nature of cancer, we conducted sensitivity analysis (model 3) by minimizing indolent HCC around the inception point by exclusion of observation period. The first 12 months of observation after the diagnosis of HPV were excluded, eliminating all cases of HCC having occurred within the first 12 months during follow-up. We conducted further sensitivity analysis (model 4 and 5) by IPTW.

### 2.9. Subgroup Analysis

We conducted subgroup analyses to examine the potential interaction of gender, age, alcohol-related illness, cirrhosis, and liver decompensation status among CHC patients. We determined significance of interaction by the likelihood ratio test.

Statistical analysis was performed by using SAS, version 9.4 (SAS Institute, Inc., Cary, NC, USA).

### 2.10. Patient Involvement Data Availability Statement

The data source used in this study was the claims data of NHIRD published by Taiwan National Health Insurance. Participants were not involved in the retrospective secondary cohort study. For Taiwan legal restrictions according to “Personal Information Protection Act”, data in this study cannot be made publicly available. Requests for data can be sent as a formal proposal to the NHIRD.

## 3. Results

### 3.1. Demographic Characteristics and Comorbidities

In total, 9905 adult CHC patients were enrolled between 2000 and 2016. Among them, we identified the HPV group (*N* = 1981) and non-HPV control group (*N* = 7924). We selected controls based on strict criteria of the same age (without age range), same sex, same inception point (HPV diagnosis date as the inception point for case and matched non-HPV), same comorbidities, and medications distribution.

Table 1 shows the characteristics of patients in both groups. In the HPV group, 50.8% of the participants were male, and 66.1% of them were between the ages of 20 and 64 years. The mean (SD) age was 57.4 (14.2) years. The most common comorbidities in the study group were peptic ulcer disease, hypertension, and hyperlipidemia. No difference in the use of aspirin, metformin, statin, NSAIDs, and interferon was shown at baseline between groups.

### 3.2. Primary Analysis

**Figure 1:**
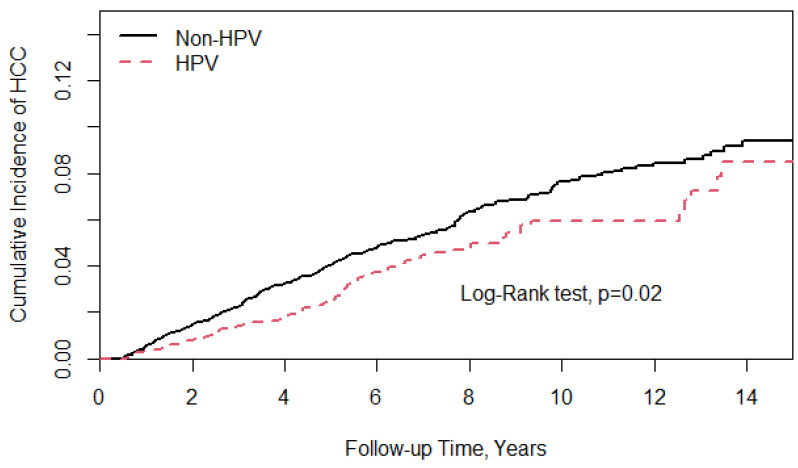
Kaplan–Meier curves showing the cumulative incidence of HCC was lower in the HPV group than in the controls (log-rank test, *p* = 0.02).

In the main model, CHC patients with HPV infection had a lower risk of developing HCC compared with those without HPV infection, 76 and 326 patients having developed HCC events, respectively. The study group had a lower incidence rate (IR) of HCC (IR, 5.69/1000 person years) than the controls (IR, 7.26/1000 person years). Figure 1 shows the Kaplan–Meier curves, showing that the cumulative incidence of HCC was lower in the HPV group than in the controls (log-rank test, *p* = 0.02). In Table 2, after a full adjustment for demographics, age, comorbidities, and medication, the aHR of HCC for the study group relative to the controls was 0.74 (95% confidence interval (CI), 0.58–0.96; *p* < 0.05). The study group’s mean time from the inception point to the development of HCC was 6.75 years (standard deviation (SD) 4.15), while in the control group it was 5.88 years (SD 4.05).

### 3.3. Sensitivity Analyses

Table 2 also shows the results of the sensitivity analysis (model two). A lower risk of HCC remained in the HPV group compared to the matched controls (aHR, 0.76; 95% CI, 0.59–0.98).

Table 3 shows the results of the sensitivity analysis (model three) (i.e., excluding HCC which occurred 12 months within the inception point), where the aHR was 0.74 (95% CI, 0.57–0.97). This model aimed to mitigate underdiagnosed HCC cases around the inception point.

Table 4 shows the results of the sensitivity analysis (models four and five), where the aHR was 0.74 (95% CI, 0.65–0.86) and 0.76 (95% CI, 0.66–0.87), respectively.

### 3.4. Subgroup Analysis

**Figure 2:**
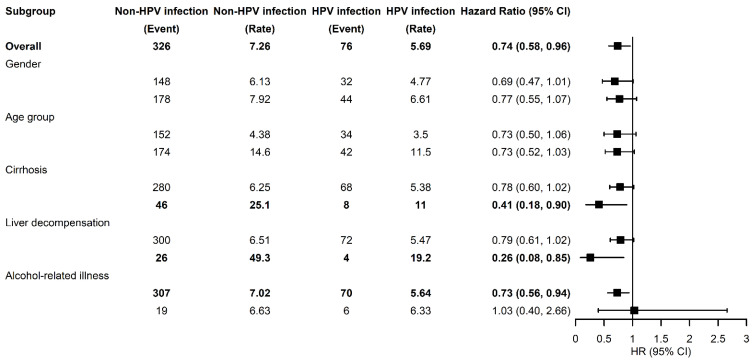
Forrest plot for reporting the subgroup analyses.

Table 5 and Figure 2 illustrate the risks of HCC stratified by the gender, age, and relevant comorbidities in CHC patients with HPV infection compared to controls. No significant interaction was found in all subgroups. CHC patients with HPV infection did not have an increased risk of HCC, including both genders and all age groups. After adjusting for sex, age, comorbidities, and medications, HPV was associated with a reduced risk of HCC in CHC patients with underlying cirrhosis (aHR, 0.41; 95% CI, 0.18–0.90), and in CHC patients with underlying liver decompensation (aHR, 0.26; 95% CI, 0.08–0.85).

Table 6 shows this association stratified by the follow-up time. A significant lower risk of HCC was found in the study group during 1–3 years and 3–5 years after the inception point (aHR, 0.61; 95% CI, 0.37–0.99; aHR, 0.55; 95% CI, 0.31–0.97, respectively).

## 4. Discussion

The current study using countrywide secondary claim data reported that a prior medically recorded HPV diagnosis was associated with a reduced risk of HCC in CHC participants. We extracted 10,800 CHC patients from an NHIRD sub-database of 200 million participants. In Taiwan, the prevalence of HCV infection among adults aged over 20 years old was about 4.4% [27]. A potential causality could be inferred by the consistent findings of risk reduction in the subgroup analyses. There was a lower risk for both sexes and all age groups which almost achieved statistical significance, although the HCC event number was small. HPV infection was an independent factor with a protective association with the HCC development after an adjustment of the baseline demographics, comorbidities, and medications.

Furthermore, in the CHC population with a baseline status of cirrhosis and liver decompensation, the HPV associated risk reduction in HCC remained. On the contrary, in the subgroup of alcohol-related disease, the negative association between HPV with HCC was obscured. The results of our epidemiological study may have potential implications for the prevention of HCC in the CHC population.

The administration of interferon with ribavirin was the treatment of choice for HCV infection over the past 20 years, which was replaced by direct-acting antiviral (DAA) agents in the last decade. To date, several studies proved the usefulness of HCC prevention by interferon-based therapy in patients with HCV [28,29]. On enrolment, we matched the use of interferon in both groups, which could minimize the influence of HCC, lowering risks through an interferon-induced sustained viral response (SVR). Moreover, DAA agents also had some positive results on HCC prevention [30,31]. However, DAA agents were not covered by the NHI in Taiwan until 2017. Even though some HCV patients may take DAAs on their own financial support and are not registered in the database, by the estimation of the Taiwan Hepatitis C Policy Guideline, this number is small and, thus, the influence could be ignored.

The underlying mechanism to explain this inverse association remains unclear. Some studies have shown that HPV 16 DNA and HPV 18-related nucleotide sequences in HCC specimens [32] and HPV 18 E6 and E7 genes can be integrated into the human hepatoma-derived cell line, Hep G2 [33]. One study even provided evidence regarding the significant association between HCV and HPV in candidates for liver transplantation [34]. A recent study [9] discussing the protein interaction network between HPV and human beings revealed that different human proteins have different numbers of interactions with HPV viral proteins. In the study, the KEGG (Kyoto Encyclopedia of Genes and Genomics) pathway analysis of human proteins showed that the gene set of the cell cycle was most actively involved with the interaction concerning HPV viral proteins, followed by viral carcinogenesis, and p53 signaling. HCV was also found to be involved in HPV interactions, but the intensity of its involvement was at the 7th position of all human proteins.

The interaction between HPV and HCV in oncogenesis has been mentioned; in a 2016 case–control study of head and neck cancers, HCV was found to be associated with HPV-positive head and neck cancers, and a possible synergistic oncogenic role of HPV E6 protein in the development of head and neck cancers has been postulated [11]. HCV and HPV seem to share oncogenic pathways in head and neck cancer; however, their interaction on the HCC development remains unclear. The role of HPV in interfering with hepatocarcinogenesis may be related to the involvement of a Toll-like receptor (TLR). Single-nucleotide polymorphisms in TLR genes may be key markers of an early susceptibility to various cancers, including HCC [35]. Since they initiate intracellular signaling pathways to induce antiviral mediators, they have been considered as the first line of antiviral immunity [36]. Studies have shown that TLR4 rs4986790 was significantly negatively associated with HCV infection [35]. Individuals carrying the rs1927911 heterozygous genotype had a significantly reduced risk of HCC [37]. On the other hand, HPV 16/18 infection was shown to be associated with TLR4 rs4986790 and rs1927911 [38], which may somewhat indicate its negative role in the development of HCV-related HCC.

Several rigorous statistical models were adopted in our study. By linking two databases (NHIRD and RCIPD) in Taiwan, the hard outcome of HCC was reliable. We found an inverse association between HPV infection and HCC. Whether there is a casual relationship between being free of HPV infection and HCC development needs further studies to clarify.

### Limitations

First of all, although the numbers enrolled in this study were adequate, the HCC event rates were lower in this study. This was more obvious when we performed the subgroup analyses. However, this pilot study still offered a new point of view of the viral interaction and the association between virus and tumor occurrence. A larger-scale cohort study could be conducted in the future.

Second, this was not a prospective study. Although we matched potential confounders between the two study groups, there was still a possibility of observation or selection bias. Fortunately, with a large number of well-validated coding for the exposure and outcome covariates, this study was able to overcome the possible bias and, thus, elucidate a clinically meaningful association.

Third, some patients might have had a recurrent HPV infection during the study period. Our study mimicked an intention-to-treat analysis; therefore, patients with another HPV infection may have been included, and, therefore, the assessment of HPV and HCC risk should be conservative.

Fourth, although both interferon and DAA reduce the risk of HCC [39], DAA was not collected in the Taiwan Insurance Administration at the time of our case recruitment, and interferon use in both groups was matched at baseline, so the bias caused by different drug treatments for HCV was minimal. Moreover, HPV vaccination may alter the immune system of the host. However, because HPV vaccination was not covered by the health insurance plan during the study period, information on HPV vaccination was not available in our database.

## 5. Conclusions

In this large-scale, retrospective CHC cohort study in Taiwan, people with a new medical record of HPV infection had a lower risk of HCC compared to CHC patients without HPV infection.

## Figures and Tables

**Table 1:** Baseline characteristics of CHC patients with and without HPV matched by age, sex, comorbidity, and medications.

	HPV Infection				
	No *N* = 7924		Yes *N* = 1981		
	*n*	%	*n*	%	*p*-Value
Age, years					0.71
20–49	2418	30.5	587	29.6	
50–64	2881	36.4	723	36.5	
≥65	2625	33.1	671	33.9	
Mean ± SD ^a^	57.4	14.5	57.4	14.2	0.84
Gender					0.32
Women	4000	50.5	975	49.2	
Men	3924	49.5	1006	50.8	
Urbanization *					0.62
1	4060	51.2	1016	51.3	
2	3129	39.5	768	38.8	
3	665	8.39	174	8.78	
4	70	0.88	23	1.16	
Individual Monthly income (TWD)					0.50
0–14,999	1541	19.5	408	20.6	
15,000–29,999	3787	47.8	938	47.4	
≥30,000	2596	32.8	635	32.1	
Comorbidity					
Hypertension	3677	46.4	933	47.1	0.58
Diabetes	1518	19.2	397	20.0	0.37
Hyperlipidemia	2947	37.2	762	38.5	0.29
Chronic kidney disease	600	7.57	156	7.87	0.65
Peptic ulcer disease	4582	57.8	1142	57.7	0.89
Helicobacter pylori infection	165	2.08	40	2.02	0.86
COPD	1772	22.4	474	23.9	0.14
Cirrhosis	452	5.70	119	6.01	0.60
Liver decompensation	164	2.07	42	2.12	0.89
Alcohol-related illness	625	7.89	173	8.73	0.22
Autoimmune disease	125	1.58	36	1.82	0.45
Medications					
Aspirin	455	5.74	125	6.31	0.34
Metformin	143	1.80	38	1.92	0.74
Statin	241	3.04	64	3.23	6.66
NSAIDs	170	2.15	41	2.07	0.83
Interferon	739	9.33	197	9.94	0.40
Outcome					
HCC	326	4.11	76	3.84	0.33

* The urbanization level was categorized by the population density of the residential area into 4 levels, with level 1 as the most urbanized and level 4 as the least urbanized. Chi-square test, ^a^ *t*-test. Abbreviations: TWD, new Taiwanese dollar; COPD, chronic obstructive pulmonary disease; NSAIDs, non-steroid anti-inflammatory drugs.

**Table 2:** Overall incidence of HCC (per 1000 person years) and estimated hazard ratios according to disease status and matching status by Cox method.

	HPV Infection	
Variable	No	Yes
Main model: Matched by age, sex, index date, low income, living area, and comorbidities		
Person years	46,626	13,364
Follow-up time (y), mean ± SD	5.88 ± 4.05	6.75 ± 4.15
Event, *n*	326	76
Rate	7.26	5.69
cHR (95% CI)	1 (reference)	0.81 (0.63, 1.04)
aHR (95% CI) ^a^	1 (reference)	0.74 (0.58, 0.96) *
Model 2: Matched by age, sex, index date, low income, living area, comorbidities, and medications		
Person years	46,701	13,364
Follow-up time (y), mean ± SD	5.89 ± 4.03	6.75 ± 4.15
Event, *n*	320	76
Rate	6.85	5.69
cHR (95% CI)	1 (Reference)	0.83 (0.64, 1.06)
aHR (95% CI) ^a^	1 (Reference)	0.76 (0.59, 0.98) *

^a^ Adjusting for age, gender, comorbidities, and medications; cHR, crude hazard ratio; aHR, adjusted hazard ratio; * *p* < 0.05.

**Table 3:** Overall incidence of HCC (per 1000 person years) and estimated hazard ratios according to disease status and matching status by Cox method (excluding HCC occurrence within 1 years after the index date).

	HPV Infection	
Variable	No	Yes
Model 3: Matched by age, sex, index date, low income, living area, and comorbidities		
Person years	46,206	13,301
Event, *n*	292	70
Rate	6.32	5.26
cHR (95% CI)	1 (reference)	0.82 (0.64, 1.07)
aHR (95% CI) ^a^	1 (reference)	0.74 (0.57, 0.97) *

^a^ Adjusting for age, gender, comorbidities, and medications; cHR, crude hazard ratio; aHR, adjusted hazard ratio; * *p* < 0.05.

**Table 4:** Estimated hazard ratios according to disease status matched by inverse probability of treatment weights.

	HPV Infection	
Variable	No	Yes
Model 4: Matched by age, sex, index date, low income, living area, and comorbidities		
cHR (95% CI)	1 (reference)	0.80 (0.69, 0.92) **
aHR (95% CI) ^a^	1(reference)	0.74 (0.65, 0.86) ***
Model 5: Matched by age, sex, index date, low income, living area, comorbidities, and medications		
cHR (95% CI)	1 (reference)	0.80 (0.70, 0.92) **
aHR (95% CI) ^a^	1 (reference)	0.76 (0.66, 0.87) ***

^a^ Adjusting for age, gender, comorbidities, and medications; cHR, crude hazard ratio; aHR, adjusted hazard ratio; ** *p* < 0.01, *** *p* < 0.001.

**Table 5:** Subgroup analysis (incidence of HCC among CHC patients with and without HPV infection).

	Non-HPV Infection			HPV Infection			Crude		Adjusted	
Variable	Event	Person Year	IR	Event	Person Year	IR	HR (95% CI)	*p*-Value	HR (95% CI)	*p*-Value
Gender										
Female	148	24,145	6.13	32	6710	4.77	0.77 (0.53, 1.13)	0.18	0.69 (0.47, 1.01)	0.06
Male	178	22,480	7.92	44	6654	6.61	0.84 (0.61, 1.17)	0.30	0.77 (0.55, 1.07)	0.12
*p* for interaction										0.76
Age (year)										
20–64	152	34,716	4.38	34	9708	3.50	0.79 (0.55, 1.15)	0.22	0.73 (0.50, 1.06)	0.09
≥65	174	11,909	14.6	42	3656	11.5	0.78 (0.55, 1.09)	0.14	0.73 (0.52, 1.03)	0.07
*p* for interaction										0.62
Comorbidities										
Cirrhosis										
No	280	44,795	6.25	68	12,634	5.38	0.86 (0.66, 1.12)	0.25	0.78 (0.60, 1.02)	0.07
Yes	46	1830	25.1	8	730	11.0	0.44 (0.21, 0.95)	0.04	0.41 (0.18, 0.90)	0.03
*p* for interaction										0.10
Liver decompensation										
No	300	46,099	6.51	72	13,155	5.47	0.84 (0.65, 1.09)	0.18	0.79 (0.61, 1.02)	0.07
Yes	26	527	49.3	4	209	19.2	0.34 (0.12, 0.97)	0.04	0.26 (0.08, 0.85)	0.03
*p* for interaction										0.14
Alcohol-related illness										
No	307	43,762	7.02	70	12,416	5.64	0.80 (0.62,1.04)	0.10	0.73 (0.56, 0.94)	0.02
Yes	19	2864	6.63	6	948	6.33	0.97 (0.39, 2.42)	0.94	1.03 (0.40, 2.66)	0.95
*p* for interaction										0.72

IR, incidence rate per 1000 person years; HR, hazard ratio; CI, confidence interval; Adjusted HR: adjusted for age, sex, comorbidities, and medications in Cox proportional hazards regression.

**Table 6:** Comparisons of incidence of HCC in different follow-up time.

	Non-HPV Infection			HPV Infection			Crude		Adjusted	
Variable	Event	Person Year	IR	Event	Person Year	IR	HR (95% CI)	*p*-Value	HR (95% CI)	*p*-Value
Follow-up (year)										
<1	34	7772	4.37	6	1959	3.06	0.70 (0.29, 1.66)	0.41	0.69 (0.29,1.65)	0.41
1–3	108	12,846	8.41	19	3445	5.52	0.66 (0.40, 1.07)	0.09	0.61 (0.37, 0.99)	0.04
3–5	78	9425	8.28	14	2716	5.15	0.62 (0.35, 1.10)	0.10	0.55 (0.31, 0.97)	0.04
≥5	106	16,582	6.39	37	5243	7.06	1.12 (0.77, 1.62)	0.57	0.97 (0.67, 1.42)	0.89

IR, incidence rate per 1000 person years; HR, hazard ratio; CI, confidence interval; Adjusted HR: adjusted for age, sex, comorbidities, and medications in Cox proportional hazards regression.

## Data Availability

Data are available from the National Health Insurance Research Database (NHIRD) published by the Taiwan National Health Insurance (NHI) Bureau. Due to legal restrictions imposed by the government of Taiwan in relation to the “Personal Information Protection Act”, data cannot be made publicly available. The Longitudinal Health Insurance Database 2000 (LHID2000) was used for this study. There were about 1 million individuals randomly sampled from the Beneficiaries of the National Health Insurance Research Database (NHIRD), that comprised approximately 23.75 million individuals in NHIRD. For details of LHID2000, please visit the website: https://nhird.nhri.org.tw/en/Data_Subsets.html.
